# Lipid nanoparticle composition for adjuvant formulation modulates disease after influenza virus infection in quadrivalent influenza vaccine vaccinated mice

**DOI:** 10.3389/fimmu.2024.1370564

**Published:** 2024-04-22

**Authors:** Sonia Jangra, Alexander Lamoot, Gagandeep Singh, Gabriel Laghlali, Yong Chen, Tingting Ye, Adolfo García-Sastre, Bruno G. De Geest, Michael Schotsaert

**Affiliations:** ^1^ Department of Microbiology, Icahn School of Medicine at Mount Sinai, New York, NY, United States; ^2^ Global Health and Emerging Pathogens Institute, Icahn School of Medicine at Mount Sinai, New York, NY, United States; ^3^ Department of Pharmaceutics, Ghent University, Ghent, Belgium; ^4^ Department of Medicine, Division of Infectious Diseases, Icahn School of Medicine at Mount Sinai, New York, NY, United States; ^5^ The Tisch Cancer Institute, Icahn School of Medicine at Mount Sinai, New York, NY, United States; ^6^ Department of Pathology, Molecular and Cell-Based Medicine, Icahn School of Medicine at Mount Sinai, New York, NY, United States; ^7^ Icahn Genomics Institute, Icahn School of Medicine at Mount Sinai, New York, NY, United States; ^8^ Marc and Jennifer Lipschultz Precision Immunology Institute, Icahn School of Medicine at Mount Sinai, New York, NY, United States

**Keywords:** influenza vaccine, QIV, adjuvant, lipid nanoparticles, ionizable lipids, antibody class switching, IgG, cytokines

## Abstract

There are considerable avenues through which currently licensed influenza vaccines could be optimized. We tested influenza vaccination in a mouse model with two adjuvants: Sendai virus-derived defective interfering (SDI) RNA, a RIG-I agonist; and an amphiphilic imidazoquinoline (IMDQ-PEG-Chol), a TLR7/8 agonist. The negatively charged SDI RNA was formulated into lipid nanoparticles (LNPs) facilitating direct delivery of SDI RNA to the cytosol, where RIG-I sensing induces inflammatory and type I interferon responses. We previously tested SDI RNA and IMDQ-PEG-Chol as standalone and combination adjuvants for influenza and SARS-CoV-2 vaccines. Here, we tested two different ionizable lipids, K-Ac7-Dsa and S-Ac7-Dog, for LNP formulations. The LNPs were incorporated with SDI RNA to determine its potential as a combination adjuvant with IMDQ-PEG-Chol by evaluating the host immune response to vaccination and infection in immunized BALB/c mice. Adjuvanticity of IMDQ-PEG-Chol with and without empty or SDI-loaded LNPs was validated with quadrivalent inactivated influenza vaccine (QIV), showing robust induction of antibody titers and T-cell responses. Depending on the adjuvant combination and LNP formulation, humoral and cellular vaccine responses could be tailored towards type 1 or type 2 host responses with specific cytokine profiles that correlated with the protective responses to viral infection. The extent of protection conferred by different vaccine/LNP/adjuvant combinations was tested by challenging mice with a vaccine-matched strain of influenza A virus A/Singapore/gp1908/2015 IVR-180 (H1N1). Groups that received either LNP formulated with SDI or IMDQ-PEG-Chol, or both, showed very low levels of viral replication in their lungs at 5 days post-infection (DPI). These studies provide evidence that the combination of vaccines with LNPs and/or adjuvants promote antigen-specific cellular responses that can contribute to protection upon infection. Interestingly, we observed differences in humoral and cellular responses to vaccination between different groups receiving K-Ac7-Dsa or S-Ac7-Dog lipids in LNP formulations. The differences were also reflected in inflammatory responses in lungs of vaccinated animals to infection, depending on LNP formulations. Therefore, this study suggests that the composition of the LNPs, particularly the ionizable lipid, plays an important role in inducing inflammatory responses *in vivo*, which is important for vaccine safety and to prevent adverse effects upon viral exposure.

## Introduction

After decades of research into influenza virus vaccines, the respiratory virus is still a major global health concern, causing thousands of cases of severe medical illness in humans every year. Several licensed influenza vaccine candidates, including recombinant, inactivated, and split influenza vaccines, have been developed and eventually licensed for use in the human population (1, 2). Despite the availability of licensed vaccines, the need to update and vaccinate people every year remains a challenge as the circulating influenza viruses can escape host immunity provided by antibodies that target the immunodominant but ever-changing antigenic sites on the hemagglutination (HA) protein (3–5). Vaccination against both seasonal influenza A virus (IAV) and influenza B virus (IBV) has been effective in controlling virus-related disease severities. However, the protection provided by humoral immunity induced by these vaccines is reported as antigenically constricted and short term. Moreover, the vaccine-induced neutralizing antibody titers drop over time, rendering the immunity less effective against an antigenically different strain of virus in the subsequent seasons (6–8). Therefore, to combat the need of a seasonal vaccine, a better cost-effective approach is required in vaccine development that can provide a broader and long-term immune response that lasts for multiple seasons.

Quadrivalent inactivated vaccines (QIV) are the most commonly used influenza vaccines. They consist of two IAV and two IBV strain components (representing the Yamagata and Victoria linages) (9, 10). QIV can induce strain-specific antibody responses with high serum IgG levels *in vivo* but are poor inducers of cell-mediated immunity and, therefore, provide limited protection against antigenically drifted virus strains. Owing to the continuous acquisition of mutations in antigenic sites of the viral hemagglutination, the protective effect of currently licensed seasonal influenza virus vaccines is time confined.

Novel vaccine concepts that aim at inducing broader, long-lasting immunity against influenza virus infection are based on enhancing vaccine-induced B- and T-cell responses that can recognize multiple antigens from vaccine components, with special focus on targeting the conserved viral epitopes. While natural infection typically results in the induction of type 1 responses, characterized by Th1 and in BALB/c mice class switching to serum IgG2a antibodies to clear viral infection (11–13), inactivated split virus influenza vaccines typically induce high IgG1 levels correlating with Th2-type immune response (14–16). Therefore, many studies, including our recently published study (17), have been focusing on combining commercially available vaccines with specific adjuvants to specifically direct responses to IgG2a or IgG1, hence inducing Th1/Th2 responses (18–21). Eventually, an efficiently balanced humoral response with enhanced T-cell activation post-vaccination is desired to be protective.

Lipid nanoparticles (LNPs) are non-viral vectors that are widely used in formulating vaccines and/or adjuvants to enhance their antigenicity and improve immune responses (22, 23). LNPs have already shown promising outcomes in formulating antigen-encoding mRNA, such as SARS-CoV-2 mRNA vaccines (24). These mRNA vaccine–LNP formulations have also successfully demonstrated the role of an LNP-based vaccine platform for an efficient induction of humoral and cell-mediated immunity. Moreover, LNPs can also be used for formulating molecular adjuvants, such as RIG-I or TLR agonists, and facilitate uptake by actively phagocytosing innate immune cell subsets (25). Nevertheless, the composition of LNP is crucial to achieve the optimal uptake by innate immune cells and efficient humoral responses. A typical LNP consists of four main components: an ionizable lipid, a phospholipid, a cholesterol moiety, and a polyethylene glycol (PEG) lipid. The ionizable lipids consist of ionizable positively charged lipids that can effectively interact with negatively charged mRNA molecules. Phospholipids and cholesterol provide structural stability to LNPs and facilitate endosomal escape, thus enhancing efficient delivery of mRNA into the cytosol of cells. The PEG lipids prolong the circulation of LNPs consisting of vaccines/adjuvants in circulation by increasing their half-life. Additionally, the surface molecules of LNPs can also be modified to target specific innate immune cells and facilitate uptake for efficient antigen presentation (23, 25–27). Overall, LNPs present as an efficient *in vivo* vaccine-adjuvant delivery system.

In this study, we investigated and compared the efficiency of two LNP formulations, consisting of different ionizable cationic lipids, in inducing both humoral and cell-mediated immune responses in a mouse model receiving a single shot of QIV from the 2018–2019 influenza season, with and without adjuvants, individually or in combination. Specifically, we used an *in vitro* transcribed Sendai virus defective-interfering RNA (17, 28, 29) (SDI-RNA; a RIG-I agonist; negatively charged and hence encapsulated into LNPs) and an amphiphilic imidazoquinoline conjugate (17, 30) (IMDQ-PEG-Chol; a modified TLR7/8 agonist with enhanced safety profile and lymph node-draining properties), previously characterized and tested by our groups, as adjuvants. Vaccine-induced antibody and T-cell responses were characterized and further correlated with lung cytokine profiles and extent of protection against virus replication upon challenge with a matching strain to the H1N1 component of QIV: A/Singapore/GP1908/2015 (IVR-180). We observed adjuvant-specific differences in B- and T-cell responses, which not only were driven by the presence of different adjuvants (SDI RNA and/or IMDQ-PEG-CHOL) but also depended on the type of ionizable/cationic lipid composition of the LNPs.

## Results

### Preparation and characterization of LNPs

**Figure 1:**
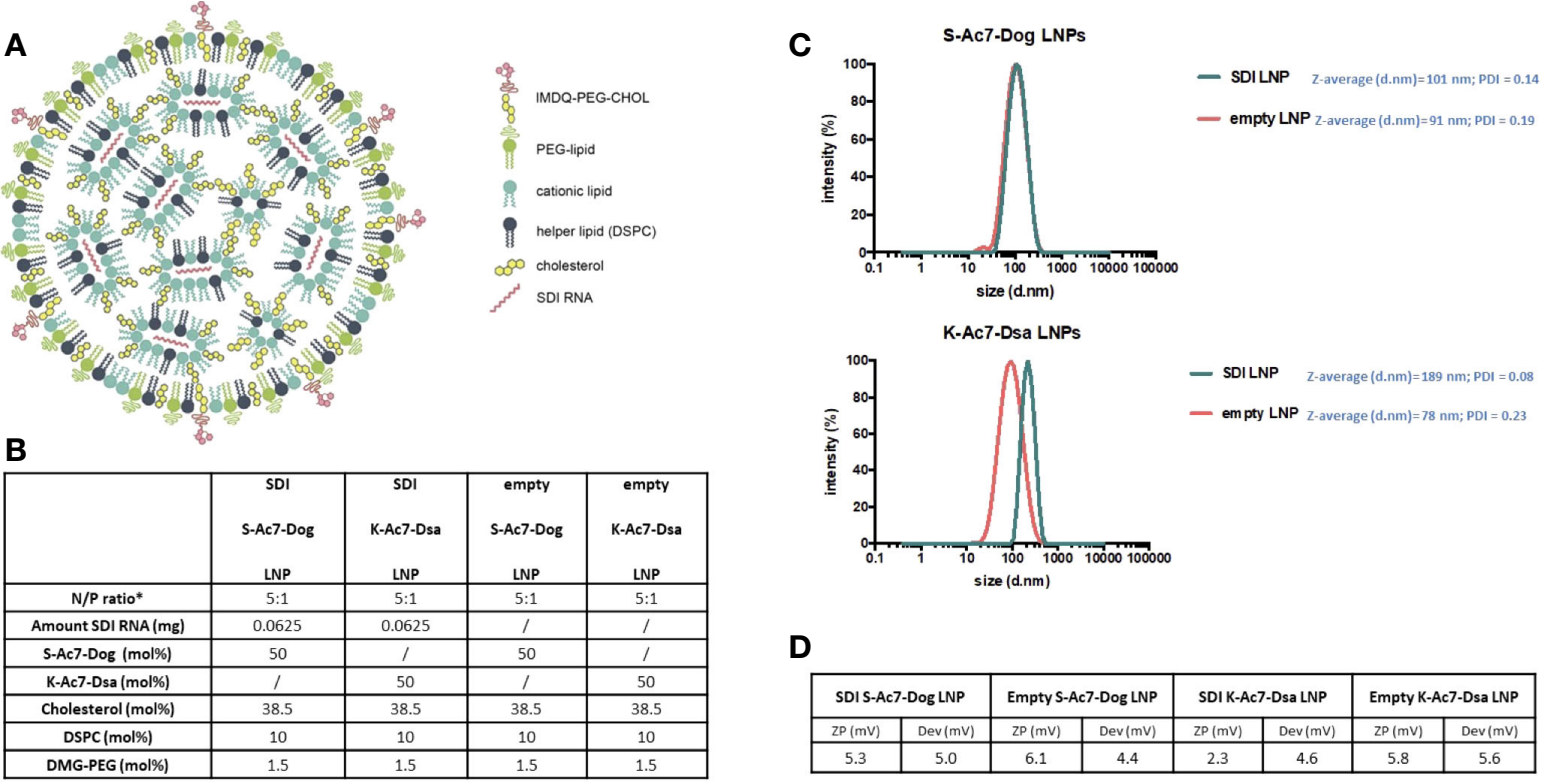
Structure and characterization of LNPs consisting of K-Ac7-Dsa or S-Ac7-Dog lipids. **(A)** Diagrammatic representation of LNP structure encapsulating SDI. **(B)** Composition ratio of different components of LNPs, with and without SDI. **(C)** Intensity-based size distribution curves measured by dynamic light scattering (DLS) of empty and SDI-incorporating S-Ac7-Dog and K-Ac7-Dsa LNP formulations. **(D)** Summary table of the LNPs zeta potential measured by electrophoretic light scattering (ELS).

Two LNP formulations were prepared by mixing an aqueous solution containing the *in vitro* transcribed SDI-RNA (or SDI) with an ethanolic solution containing (1) ionizable lipids, either K-Ac7-Dsa (comprising a ketal bond) or S-Ac7-Dog (comprising a disulfide bond; chemical structure outlined in **Supplementary Figure 1**), to interact with negatively charged SDI and mediate endosomal escape; (2) cholesterol, for structural stability; (3) dioleoylphosphatidylethanolamine (DOPE) phospholipid, as helper lipids to aid in nanoparticle formation; and (4) 1,2-distearoyl-rac-glycero-3-methylpolyethylene glycol (DSG-PEG; 2kDa PEG) to provide steric hindrance and thus avoiding aggregation and promoting mobility *in vivo* (23). The structure and composition of LNP incorporating SDI RNA is schematically represented in **Figures 1A, B**, respectively. The molar ratio of ionizable lipid (K-Ac7-Dsa or S-Ac7-Dog):cholesterol:DOPE : DSG-PEG was chosen to be 50:38.5:10:1.5, based on literature (24). Empty LNPs that did not contain SDI were prepared as control and hence referred to as empty LNP or LNP(−). Both LNPs encapsulating SDI [S-Ac7-Dog(SDI) and K-Ac7-Dsa (SDI)] and corresponding control empty LNPs [S-Ac7-Dog(−) and K-Ac7-Dsa (−)] were fabricated and characterized for their size and zeta potential (ZP) (**Figures 1C, D**). While K-Ac7-Dsa(−) and K-Ac7-Dsa(SDI) LNPs showed some differences in their size (78 and 189 nm, respectively), the S-Ac7-Dog(−) and S-Ac7-Dog(SDI) LNPs showed similar size distributions (91 and 101 nm, respectively) with low polydispersity indices (PDI < 0.25; as shown in **Figure 1C**) and a positive ZP of approximately 4–6.5 mV at physiological pH (**Figure 1D**).

### LNP formulations with adjuvanted QIV define IgG subtype profile, with S-Ac7-Dog LNPs inducing higher antibody titers than K-Ac7-Dsa LNPs

**Figure 2:**
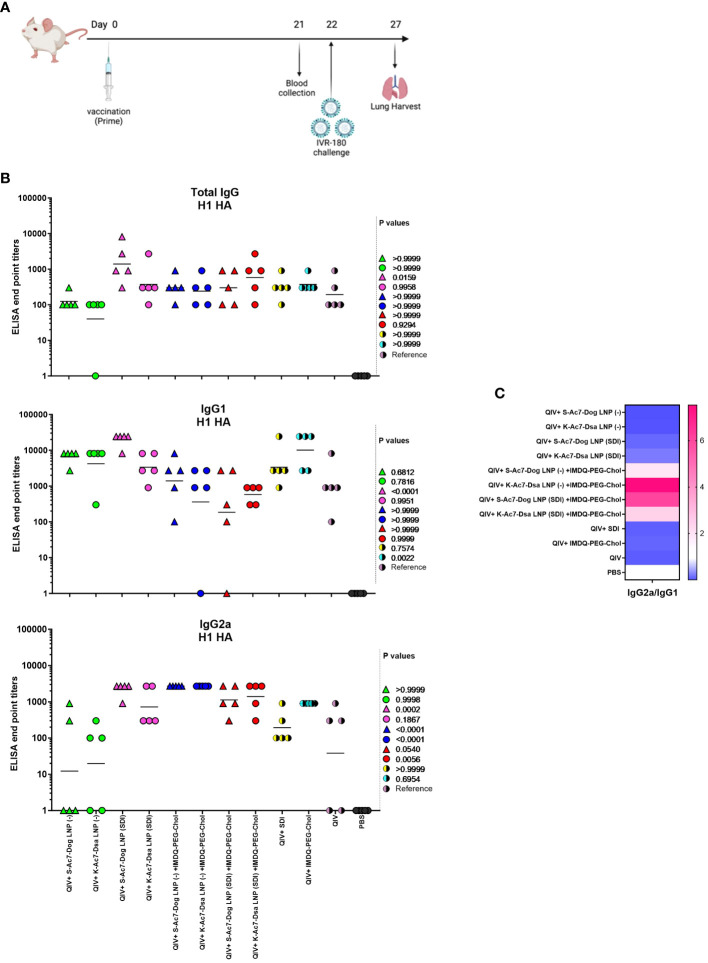
S-Ac7-Dog (− or SDI) and K-ac7-Dsa (− or SDI) in combination with IMDQ-PEG-Chol define IgG subtype profile. **(A)** Study outline. **(B)** Graphs showing ELISA end point titers calculated based on OD 450 ELISA values against serum dilutions for total IgG, IgG1, and IgG2a (*n* = 5 per group), represented as geometric mean. The statistical analysis was performed using one-way ANOVA with a Dunnett’s multiple comparison test, and the *p*-values shown are calculated in reference to the unadjuvanted QIV group, which received neither adjuvant nor LNP formulations. **(C)** Heatmap showing the ratio of end point titers of IgG2a to IgG1 and represented as geometric mean of IgG2a/IgG1 ratios for all animals in each group.

We evaluated the potential of empty and SDI-incorporating S-Ac7-Dog and K-Ac7-Dsa LNPs to adjuvant a licensed QIV, with and without combination with IMDQ-PEG-Chol adjuvant, using our previously well-established preclinical vaccination-infection animal model (17, 30). The study is outlined in **Figure 2A**. We vaccinated 6- to 8-week female BALB/c mice with unadjuvanted QIV or in combination with SDI or IMDQ-PEG-Chol, or both, while mock animals received PBS. We further tested the adjuvant effect for QIV upon co-administration of SDI and/or IMDQ-PEG-Chol combined with one of the two LNPs containing different cationic lipids (S-Ac7-Dog or K-Ac7-Dsa) as described in the previous section. LNPs either were empty (−) or had SDI incorporated. The rationale of this setup is that LNP-formulated SDI can, besides endosomes, also be delivered to the cytosol, thereby promoting more efficient RIG-I-mediated innate immune sensing. On the other hand, IMDQ-PEG-Chol is expected to incorporate efficiently in LNPs via its cholesterol moiety. The animals were vaccinated only once via the intramuscular (IM) route. The serum collected 3 weeks post-vaccination was examined for the presence of H1 HA-specific IgG antibodies using enzyme-linked immunosorbent assay (ELISA) for total IgG, IgG1, and IgG2a (**Figure 2B**).

No virus-specific antibody titers were detected in the serum from the mock PBS group. The group that received unadjuvanted QIV was used as the reference to compare the IgG responses of other groups. QIV formulated with S-Ac7-Dog LNP (−) or K-Ac7-Dsa LNP(−), corresponding to empty S-Ac7-Dog and empty K-Ac7-Dsa LNPs, respectively, showed higher IgG1 titers but the lowest IgG2a titers compared with the corresponding S-Ac7-Dog LNP(SDI) or K-Ac7-Dsa LNP(SDI) groups (**Figure 2B**; **Supplementary Figure 2**), thereby illustrating the intrinsic adjuvant effect of LNPs. Additionally, QIV formulated with SDI-incorporated LNPs [S-Ac7-Dog LNP(SDI) and K-Ac7-Dsa LNP(SDI)] can induce a balanced IgG1 and IgG2a antibody response. Overall, the total IgG levels were found similar among all adjuvanted and LNP-formulated groups post-prime vaccination. However, the S-Ac7-Dog LNP(SDI) seemed to induce slightly higher total IgG compared to K-Ac7-Dsa LNP (SDI) in the corresponding SDI ± IMDQ-PEG-Chol combination adjuvanted groups. A similar observation was made for IgG1 antibody titers with S-Ac7-Dog LNPs inducing higher IgG1 titers than respective K-Ac7-Dsa LNP groups. Consistent with our previous findings (17), IMDQ-PEG-Chol administration, with either empty or SDI-incorporated S-Ac7-Dog or K-Ac7-Dsa LNPs, skews the antibody responses towards IgG2a with a significant reduction in IgG1 titers (**Figure 2C**). QIV+SDI and QIV+IMDQ-PEG-Chol groups, with no LNP formulations, were used as control vaccination groups for the study and to correlate with our previous study.

### QIV adjuvanted with IMDQ-PEG-Chol exhibit better control over virus neutralization when formulated in S-Ac7-Dog LNPs than in K-Ac7-Dsa LNPs

**Figure 3:**
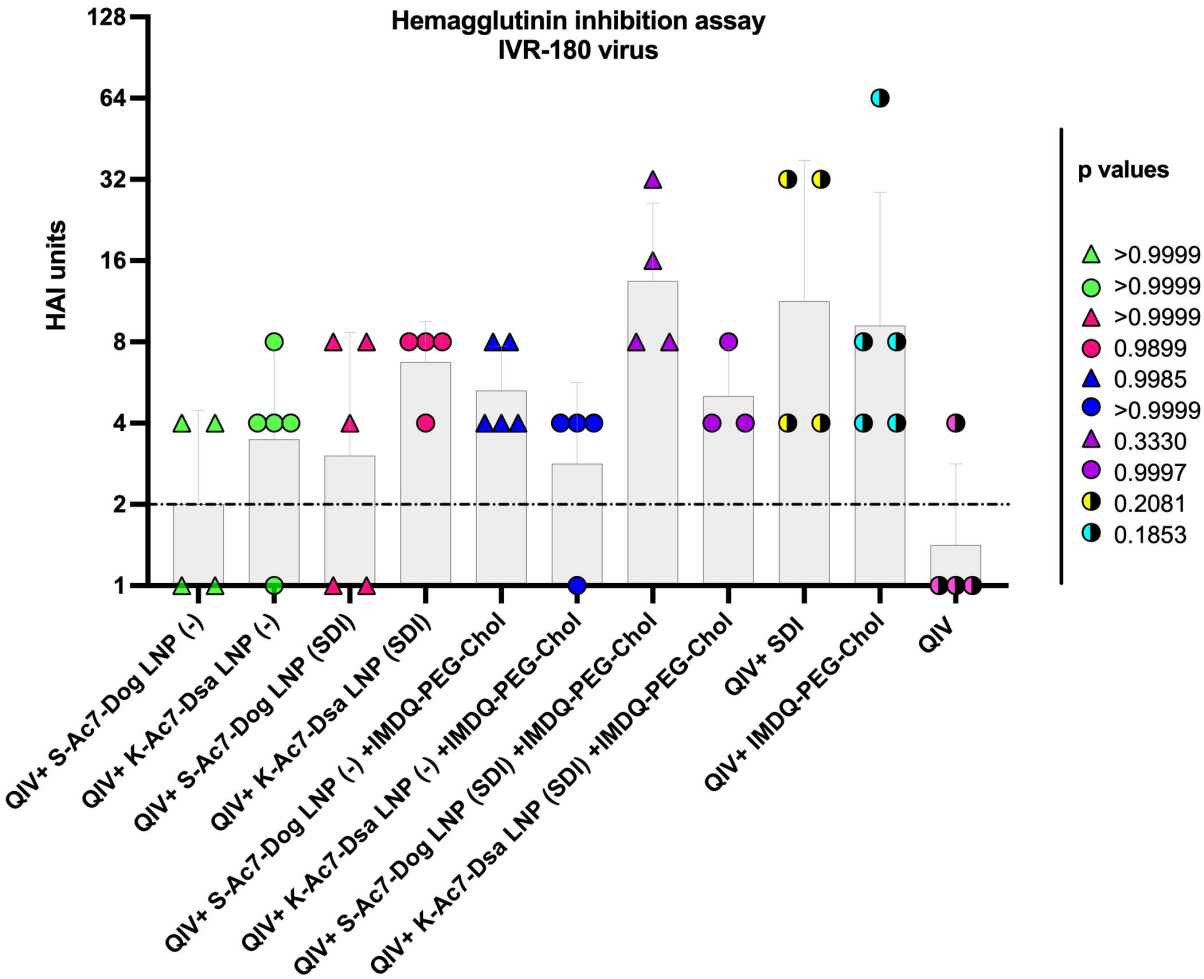
QIV adjuvanted with IMDQ-PEG-Chol exhibits a better control over virus infection when formulated in S-Kc7-Dog LNPs. The sera collected from all vaccinated animals 3 weeks post-vaccination were tested for HAI titers, using 4 HA units of the IVR-180 virus. The HAI titers are represented as geometric mean ± geometric SD for *n* = 4 animals per group. The samples with un-detectable HAI titers were set as 1 and the limit of detection was set to 2, corresponding to the lowest detectable HAI units. The statistical analysis was performed using one-way ANOVA with a Dunnett’s multiple comparison test. The *p*-values shown are calculated in reference to the QIV group, which received neither adjuvant nor LNP formulations.

Neutralizing antibodies are important defense mechanisms during viral infection. These antibodies bind to the viral antigens and change the conformation, thereby blocking the viral attachment to cells. As a surrogate for virus neutralizing antibody levels, we performed hemagglutination inhibition (HAI) assays with post-vaccination sera collected from all vaccinated animals at 3 weeks post-vaccination. As shown in **Figure 3**, the mice that received unadjuvanted QIV showed low HAI titers, which were not significantly different from the groups that received QIV formulated in either empty or SDI-containing S-Ac7-Dog or K-Ac7-Dsa LNPs. The group administered with QIV+IMDQ-PEG-Chol, without LNP formulations, showed significantly higher HAI titers compared with the unadjuvanted QIV group. HAI titers were significantly higher for the groups that received empty or SDI-containing S-Ac7-Dog in the LNP formulation when combined with the IMDQ-PEG-Chol adjuvant. However, the corresponding K-Ac7-Dsa LNP-formulated groups did not show any significant differences in HAI titers compared with the unadjuvanted or unformulated QIV group.

### QIV formulated in either SDI-containing S-Ac7-Dog or K-Ac7-Dsa LNPs efficiently induce T-cell responses when combined with IMDQ-PEG-Chol

**Figure 4:**
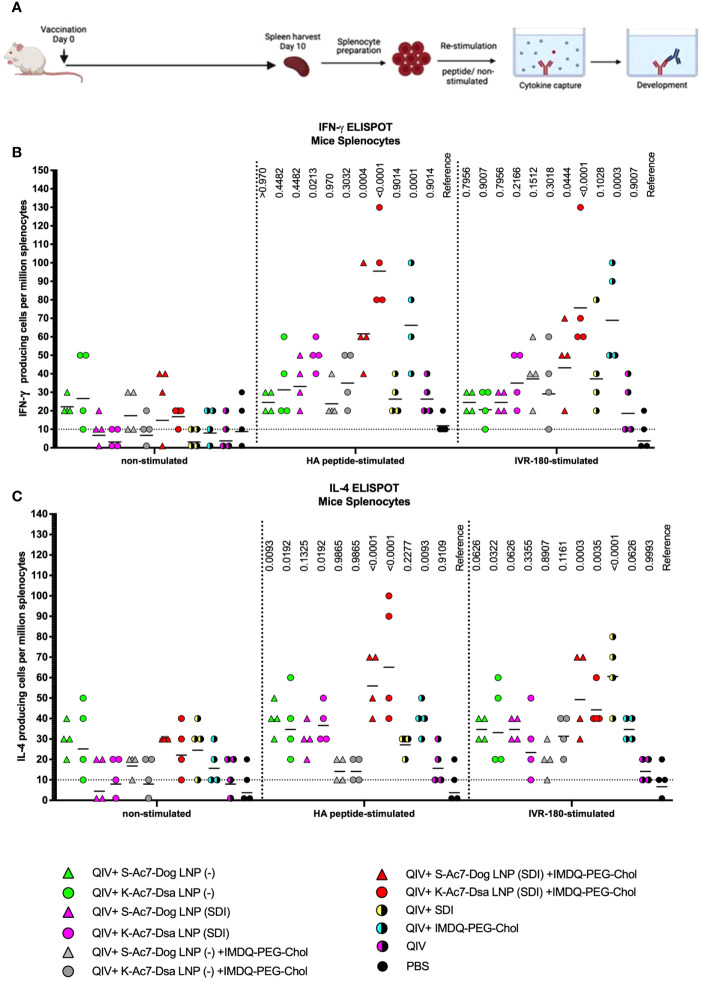
QIV formulated in S-Ac7-Dog(SDI) and K-Ac7-Dsa(SDI) LNPs efficiently induces T-cell responses when combined with IMDQ-PEG-Chol. **(A)** Experiment outline. **(B, C)** Six- to 8-week BALB/c mice were vaccinated with QIV with and without IMDQ-PEG-Chol and formulated into S-Ac7-Dog(− or SDI) or K-Ac7-Dsa(− or SDI) LNPs. The spleens were harvested at 10 DPV to examine the T-cell activation by IFN-γ **(B)** and IL-4 **(C)** ELIspots, upon restimulation with H1-HA short-overlapping peptides or live IVR-180 (A/Singapore/GP1908/2015 H1N1) virus. The results are represented as IFN-γ- or IL-4-producing cells per million splenocytes (geometric mean ± geometric SD) for *n* = 4 animals per group. The cutoff was set to 10, which indicates one spot in any well. The wells with no spots were given the value 1. The statistical analysis was performed using one-way ANOVA with a Dunnett’s multiple comparison test and the *p*-values shown are calculated in reference to the respective PBS group.

Helper CD4+ and cytotoxic CD8+ T cells play an important part in vaccination-mediated humoral and cellular responses by facilitating Ig class switch during B-cell maturation and direct killing of infected cells, respectively. T cells can recognize foreign antigens presented on the major histocompatibility complex (MHC) molecules on infected cells followed by the release of various cytokines, including IFN-γ and IL-4, two major cytokines corresponding to helper type 1 (Th1) and type 2 (Th2) T cells, respectively. IFN-γ and IL-4 can modulate class switching of B cells to IgG2a or IgG1 (19). To study the correlation between the two cytokines and antibody responses in our vaccination model, we examined the release of IFN-γ and IL-4 from splenocytes obtained from the mice 10 days post-vaccination (DPV) (outlined in **Figure 4A**), in the presence and absence of specific antigen (IVR-180 virus or H1 HA peptide) using enzyme-linked immunosorbent spot (ELISpot) assays.

As shown in **Figure 4**, antigen-specific IFN-γ (**Figure 4B**) or IL-4 (**Figure 4C**) release from the splenocytes was very low in the absence of a stimulant, except for QIV+S-Ac7-Dog(−) or QIV+K-Ac7-Dsa(−) groups, suggesting a basal level of non-specific stimulation after vaccination by the empty LNPs. Upon stimulation with specific H1-HA peptide or IVR-180 virus, the number of splenocytes producing antigen-specific IFN-γ as well as IL-4 were found to be higher in the mice that received QIV+IMDQ-PEG-Chol formulated in S-Ac7-Dog(SDI) or K-Ac7-Dsa(SDI) LNPs, suggesting an induction in both Th1 and Th2 immune response. The group administered with QIV+IMDQ-PEG-Chol, without LNP formulations, showed high IFN-γ release compared with the unadjuvanted QIV group, consistent with our previous study. Mice that received PBS were used as a reference for statistical comparison.

### S-Ac7-Dog and K-Ac7-Dsa LNP formulations with SDI and/or IMDQ-PEG-Chol potentiates QIV-mediated protection against viral challenge with homologous influenza virus

**Figure 5:**
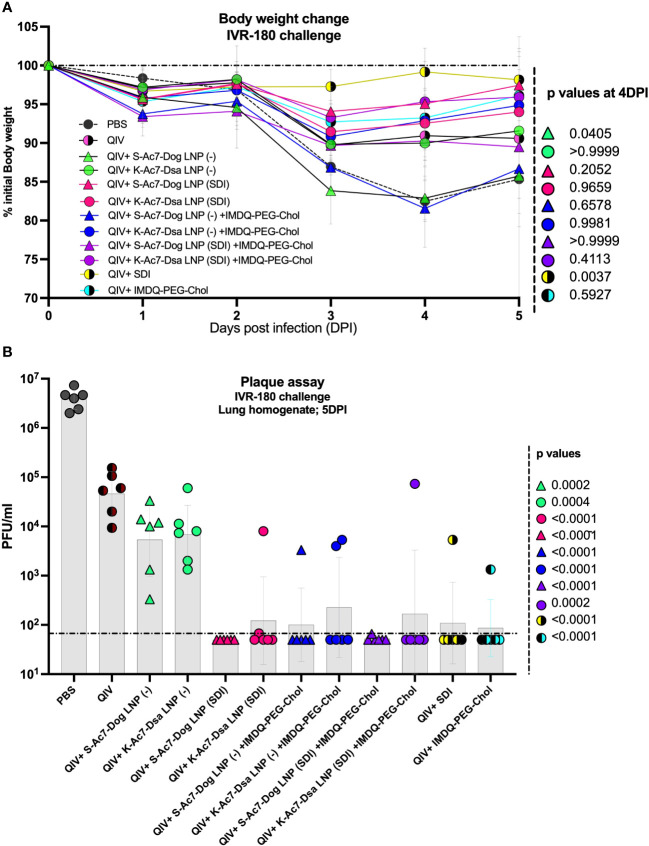
QIV formulated in S-Ac7-Dog(SDI) and K-Ac7-Dsa(SDI) LNPs contributes to reduction in IVR-180 virus replication in lungs of vaccinated animals irrespective of combination with IMDQ-PEG-Chol. All unvaccinated and QIV ± IMDQ-PEG-Chol ± S-Ac7-Dog(SDI) or K-Ac7-Dsa(SDI) vaccinated animals were intranasally challenged with 18,000 plaque‐forming units (PFU) of IVR-180 virus per animal. **(A)** The body weight of each animal in all groups was recorded every day until the day of harvest and represented as percentage of initial body weight for each group (*n* = 6) (geometric mean ± geometric SD). **(B)** The viral lung titers were quantified at 5 DPI by plaque assays on pre-seeded MDCK cells and are represented as PFU/mL for *n* = 6 animals per group (geometric mean ± geometric SD). Each data point represents one animal in the respective group. Statistical analysis was performed using one-way ANOVA with a Dunnett’s multiple comparison test. The *p*-values shown are calculated in reference to the unadjuvanted QIV group, which received neither adjuvant nor LNP formulations.

To further correlate B- and T-cell responses with the extent of protection against virus replication *in vivo*, all vaccinated and un-vaccinated mice were given a high-titer virus challenge with 18,000 plaque-forming units (PFU) of IVR-180 virus per animal. A single dose of vaccination was found effective in conferring protection from severe morbidity in challenged animals compared with mock-challenged mice in the initial 5 days of virus challenge. As shown in **Figure 5A**, the groups receiving unadjuvanted QIV and QIV with K-Ac7-Dsa(− or SDI) LNPs showed less than 10% body weight loss over 5 days post-infection. The unvaccinated PBS group lost approximately 20% body weight by 4 DPI. Interestingly, the mice vaccinated with S-Ac7-Dog (−), irrespective of the combination with IMDQ-PEG-Chol, showed drastic weight loss over 5 days, almost comparable to the unvaccinated PBS group. This is especially important to note because the same S-Ac7-Dog LNPs incorporating SDI did not show such extensive weight loss in vaccinated/challenged animals, suggesting higher morbidity in virus-infected lungs in case of empty S-Ac7-Dog LNP formulations.

Irrespective of the body weight loss differences attributed by LNPs, all groups that received adjuvanted QIV showed a lower amount of replicating virus in their lungs at 5 DPI (**Figure 5B**), compared with the unadjuvanted QIV group. QIV formulated in either of the two empty LNPs [S-Ac7-Dog(−) or K-Ac7-Dsa(−)] did not provide significant protection in the initial days of infection compared with the unadjuvanted QIV group, contrary to the higher IgG1 induction. The groups that received QIV with either S-Ac7-Dog(− or SDI) or K-Ac7-Dsa(− or SDI) LNPs, irrespective of IMDQ-PEG-Chol combination, resulted in significantly better control of lung virus replication, with very low detectable titers in their lungs, and, therefore, correlated with enhanced vaccine responses observed in mice. Interestingly, S-Ac7-Dog LNPs resulted in lower body weight loss in the first 5 days of infection, compared to K-Ac7-Dsa LNPs in animals when compared between corresponding adjuvant groups.

### Incorporation of SDI as an adjuvant improves the cytokine profile in lungs of infected animals causing less morbidity as compared to empty S-Ac7-Dog LNP formulations

**Figure 6:**
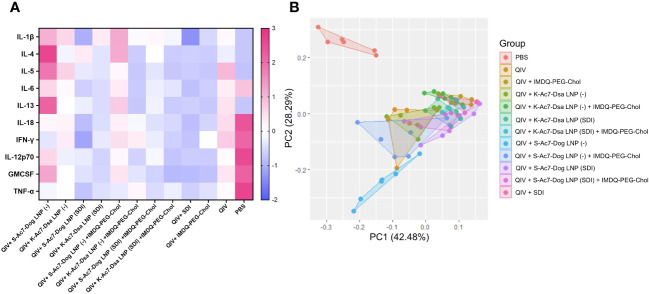
Heatmap showing cytokine profile in lungs of vaccinated mice upon intranasal challenge with IVR-180. All unvaccinated and vaccinated animals were intranasally challenged with 18,000 PFU of IVR-180 virus. The lungs were collected at 5 DPI and the cytokine levels were quantified by multiplex ELISA. Levels of IL-1β, IL-5, IL-13, TNF-α, IL-12 p70, IL-4, IL-6, IFN-γ, IL-18, and GMCSF for *n* = 6 animals per group are represented as median of *z*-scores **(A)** and PCA plots **(B)** for all animals in each group.

The body weight data over 5 DPI suggested that the administration of empty S-Ac7-Dog LNP as vaccine ± adjuvant formulation, although protective, resulted in higher body weight loss, comparable to PBS-vaccinated control mice. The lungs of all infected animals were examined for their cytokine profiles at 5 DPI. As shown in **Figure 6** ; **Supplementary Figure 3**, the cytokine profiles in the infected lungs of animals from vaccinated or unvaccinated/PBS groups were different. The PBS group showed high levels of inflammatory cytokines including IL-6, IL-18, IL-12 p70, IFN-γ, and TNF-α as well as chemokine GMCSF, which may suggest enhanced vascular permeability and increased infiltration of innate immune cells in response to infection in unvaccinated animals. As shown in **Figure 6A**, these cytokine levels were significantly lower in all vaccinated groups, implying a better control over inflammation and morbidity in response to the virus. Some of the cytokines were very low in the PBS group, especially the signatures for T-cell responses such as IL-4, IL-5, and IL-13, which is in line with the typical type 1 skewed host immune response to influenza infection. Interestingly, pro-inflammatory cytokines and chemokines, including IL-1β, GMCSF, and the type 2 cytokines IL-4, IL-5, IL-6, and IL-13, were significantly elevated in unadjuvanted QIV and QIV± IMDQ-PEG-Chol formulated with empty LNPs. This was not the case for the corresponding groups with LNP(SDI), especially for those LNP groups with S-Ac7-Dog lipids. The PCA plot in **Figure 6B** clusters different vaccinated and unvaccinated groups based on their differences in the production of inflammatory cytokines and chemokines in lungs post-infection. The unvaccinated PBS group clusters far away from all vaccinated animals, corresponding to high inflammation and chemokine production in lungs post-infection with lower interleukins. Moreover, the QIV+ S-Ac7-Dog(−) vaccinated group clustered separately from the other vaccinated groups. Additionally, the group that received QIV+ S-Ac7-Dog(−) combined with IMDQ-PEG-Chol is clustered together with unadjuvanted QIV, suggesting that the use of empty S-Ac7-Dog is disadvantageous as it reduces the protective effect of IMDQ-PEG-Chol when used without any lipid formulation. The other vaccinated groups showing low inflammation in lungs are clustered together.

## Discussion

QIV, consisting of two IAV and two IBV strain components, are one of the licensed influenza vaccines, and require annual updates to provide immunity against circulating influenza viruses in the human population. Several studies have focused on improving split vaccines’ efficiency by combining them with adjuvants, including our recent study on SDI RNA and IMDQ-PEG-Chol (RIG-I and TLR7/8 agonists, respectively, and both potent inducers of innate immune responses), as adjuvants for QIV. Then again, the stability of these vaccines ± adjuvants, including SDI RNA, and their efficient *in vivo* delivery are still challenging. To overcome this, LNPs have emerged as promising vehicles for *in vivo* delivery systems. Their ability to encapsulate, stably carry, and efficiently deliver the molecules of interest has provided an effective platform in pharmaceutical and vaccine fields. One such example is highly efficient current mRNA vaccines for SARS-CoV-2 that use LNP-based formulations (24). Nevertheless, these vaccines still need multiple booster doses to be sufficiently effective against emerging virus strains and therefore are limited in inducing an antigenically broader immune response. Therefore, optimization of LNP dose and composition needs to be tailored together with vaccine and/or adjuvant combinations in order to get the desired vaccination outcomes in terms of both humoral and cellular responses, as well as providing protection against viral infections (22, 23).

In this study, we tested adjuvant formulations for QIV [2018–2019 season; with A/Singapore/GP1908/2015 IVR-180 (H1N1) as one of the two IAV components] with two different LNP formulations using either S-Ac7-Dog or K-Ac7-Dsa cationic lipids. Since the lipids are positively charged, they can stably interact with negatively charged biomolecules. The vaccine–LNP combinations were further adjuvanted with either one or both of SDI-RNA and IMDQ-PEG-Chol to explore the outcomes of the vaccine ± adjuvant ± LNP formulations in the context of antibody responses, antibody class switching, T-cell responses, protection against *in vivo* IVR-180(H1N1) virus challenge, and inflammatory responses in the infected lungs.

The unadjuvanted QIV vaccine induces modest serum IgG1 and IgG2a titers as well as low HAI titers 3 weeks after a single dose of intramuscular vaccination in mice. This is consistent with our previous findings using the same mouse vaccination model (17). The low antibody titers for unadjuvanted QIV correlated well with low T-cell responses (IL-4 and IFN-γ ELIspots) as well as inadequate protection against virus infection, implied by high levels of replicating virus in lungs upon intranasal IVR-180 challenge. These animals also showed the most body weight loss and high levels of inflammatory cytokines in their lungs post-infection, suggesting the recruitment of immune cells to aid in the control/clearance of the virus. QIV formulated with either of the two LNPs and/or further combined with either SDI and/or IMDQ-PEG-Chol as adjuvant show a boosted total IgG response and a better control over virus replication by 5 DPI. However, the administration of individual or combination adjuvants skewed the B-cell class switch as well as T-cell responses in vaccinated mice. Upon a single vaccination, SDI induced a balanced IgG1/IgG2a response, IMDQ-PEG-Chol directed more towards an IgG2a response, and the combination of the two skewed completely towards IgG2a, when formulated into S or K LNPs, suggesting very strong class switching events driven by this combination of adjuvants and consistent with our previous findings. Interestingly, the combination of the two adjuvants SDI and IMDQ-PEG-Chol induces a balanced type I and II T-cell response as suggested by both IL-4 and IFN-γ release upon antigenic restimulation. Despite the fact that the combination of IMDQ-PEG-Chol and SDI in LNP formulations results in a balanced Th1/Th2, a very strong type 1 skewing driven by IgG2a is seen when antibody responses are considered.

Interestingly, S-Ac7-Dog LNPs were found to be more immunogenic than K-Ac7-Dsa LNPs in inducing both humoral and cellular responses in the corresponding groups. This can also be explained by the smaller size of S-Ac7-Dog LNPs than K-Ac7-Dsa LNPs, as the size of LNPs plays an important role in vaccine efficacy (31). Yet, vaccination with empty S-Ac7-Dog LNPs resulted in lack of protection from morbidity in challenged mice. Remarkably, S-Ac7-Dog(−) + IMDQ-PEG-Chol vaccination also resulted in lack of protection from morbidity after infection, with body weight loss comparable to unvaccinated/PBS control animals. This lack of protection from morbidity was accompanied by enhanced inflammatory cytokine responses including interleukins IL-4, IL-5, IL-6, and IL-13, interferon gamma (IFN-γ), as well as chemokine GMCSF. In contrast, when S-Ac7-Dog LNPs were combined with SDI, inflammation is relatively reduced in infected lungs as suggested by the chemokine/cytokine levels. The differences might be attributed to the lower stability of S-Ac7-Dog than K-Ac7-Dsa lipids in the respective LNPs, which might be stabilized by the addition of oppositely charged SDI molecules. However, additional experiments would be needed to confirm this.

Overall, we compared two different lipid compositions in LNP formulations, empty or loaded with individual or combination adjuvants. Different combinations affected both B- and T-cell responses along with vaccine/adjuvant/LNP-dependent inflammation in single vaccinated mice upon virus infection. The negatively charged SDI might stably interact with cationic lipid moieties, providing more stability to the entire structure and thereby reducing inflammation in infected animals. The immunogenicity and protection data in mice combined with the cytokine/chemokine induction indicate that lipid composition of LNPs used in vaccines is important and can skew host immune responses to subsequent infection, and therefore is important for vaccine safety and efficacy.

## Methods and reagents

A list of reagents used in the study is provided in **Supplementary Table 1**.

### Cell lines

The Madin-Darby canine kidney (MDCK) cell line was maintained in Dulbecco’s Modified Eagle Medium (DMEM) supplemented with 10% fetal bovine serum (FBS) and 1× antibiotics (penicillin/streptomycin).

### QIV vaccine

Quadrivalent inactivated influenza vaccine (FLUCALVEX 2018/2019 season Lot 252681) was obtained from Seqirus. The vaccine consists of MDCK-grown two IAV and two IBV- A/Singapore/GP1908/2015 IVR-180 (H1N1) (A/Michigan/45/2015-like virus), A/North Carolina/04/2016 (H3N2) (A/Singapore/INFIMH-16-0019/2016-like virus), B/Iowa/06/2017 (B/Colorado/06/2017-like virus), and B/Singapore/INFTT-16-0610/2016 (B/Phuket/3073/2013-like virus).

### SDI-RNA

The SDI-RNA (or SDI) was *in vitro* transcribed as described in our recent study (17).

### Lipid nanoparticle fabrication

SDI or SDI equivalent (1 μg) was encapsulated in LNPs by rapid mixing under vigorous stirring of an acetate buffer (5 mM, pH 4.5) containing SDI with an ethanolic solution containing the ionizable lipid S-Ac7-Dog or K-Ac7-Dsa (to obtain S-Ac7-Dog and K-Ac7-Dsa LNPs respectively), cholesterol, 1,2-dioleoyl-n-glycero-3-phosphoethanolamine (DOPE), and a poly(ethylene glycol)-lipid (DSG-PEG; PEG had an MW of 2 kDa) at a 50:38.5:10:1.5 ratio. After mixing, LNPs were dialyzed against 1× PBS to get rid of ethanol and the pH was adjusted to 7.4. An N/P (ionizable nitrogen atoms of the ionizable lipid to anionic phosphor atoms of SDI) molar ratio of 5:1 was targeted.

### LNP characterization

The diameter and polydispersity index (PDI) of LNPs were measured with dynamic light scattering (DLS) at physiological pH. Each sample was measured in triplicate and a cumulative average of *z* average and PDI was calculated. For ELS, each sample was appropriately diluted in HEPES buffer and measurements were taken in triplicate. The Zeta potential was calculated for all samples based on the Smoluchowski equation.

### Vaccine-adjuvant preparation and administration

For each animal, 1.5 mg of HA equivalent of QIV was mixed with empty or SDI-encapsulating S-Ac7-Dog or K-Ac7-Dsa LNPs, with or without 100 μg of IMDQ-PEG-Chol (equivalent to 10 μg of core IMDQ), and vortexed for 30 s. Adjuvant doses were chosen based on our previously published work with these adjuvants (17, 30). Unadjuvanted or adjuvanted QIV, with or without LNP formulations, was administered intramuscularly in a total of 50 μL per mouse, in the right hind leg. The control group was administered with equal volume of PBS instead of vaccine or vaccine ± adjuvant ± LNP mixture. All animals received only one dose of vaccine without any further boosters.

### IVR-180 virus

A/Singapore/GP1908/2015 IVR-180 (H1N1) was grown in 8-day-old embryonated chicken eggs and was titrated by plaque assay on pre-seeded MDCK cells.

### Mouse model for vaccination

The study was performed on 6- to 8-week-old female BALB/c mice strains obtained from Jackson Laboratories, CT. The mice were housed with food and water *ad libitum* in a pathogen-free facility at Icahn School of Medicine at Mount Sinai, New York. All mice were vaccinated intramuscularly (50 μL; hamstring muscles of the left hind leg; per mouse) and infected intranasally (in 50 μL total volume per mouse) under ketamine/xylazine anesthesia. All procedures were performed according to the protocols approved by the Icahn School of Medicine at Mount Sinai Institutional Animal Care and Use Committee (IACUC-PROTO202100007).

### Serum collection for serology

Mice blood was collected by submandibular bleed 3 weeks post-vaccination from all animals. The blood was allowed to clot at 4°C for overnight. The serum was collected after a brief centrifugation and was heat inactivated at 56°C for 30 min. The samples were stored at −20°C until further use.

### Enzyme-linked immunosorbent assay

ELISA was performed to quantify the vaccine-specific IgG titers in mice sera. Briefly, ELISA plates were coated with recombinant trimeric HA derived from the A/Michigan/45/2015 H1N1 virus, which is closely related to IVR-180, as previously described (17), equivalent to 2 µg H1N1-HA/mL, in bicarbonate buffer and left overnight at 4°C. Plates were washed three times with 1× PBS and incubated in 100 μl of blocking buffer per well [5% fat-free milk in PBST (1× PBS + 0.1% Tween20)] for 1 h at room temperature (RT). In the meantime, the serum samples were serially diluted 3-fold, starting with 1:100 dilution, in blocking buffer and 50 μL of each sample dilution was incubated on HA-coated ELISA plates overnight at 4°C. The following day, the plates were washed three times with PBST and incubated with 100 μL of diluted horseradish peroxidase (HRP)-conjugated anti-mouse secondary total IgG (1:5,000) or IgG1 (1:2,000) or IgG2a (1:2,000) antibodies, for 1 h at RT. Finally, the plates were washed three times in PBST and incubated with 100 µl of tetramethylbenzidine (TMB) substrate at RT until the blue color appeared. The reaction was terminated with 50 µl of 1 M sulfuric acid (H_2_SO_4_), and the absorbance was measured at 450 nm and 650 nm wavelengths using BIOTEK ELISA plate reader.

### Hemagglutination inhibition assay

Mice sera collected 3 weeks post-vaccination were treated with four volumes of receptor destroying enzyme (RDE) at 37°C overnight, followed by treatment with five volumes of 1.5% sodium citrate at 56°C, 30 min. The thus obtained 1:20-diluted serum samples were further serially diluted in a transparent V-bottom 96-well plate and incubated with 4 HA units per well of IVR-180 virus for 30 min at RT, followed by the addition of 0.5% chicken red blood cells for 40 min at 4°C. The results were recorded as HAI titers.

### Enzyme-linked immunosorbent spot

Mice were vaccinated with different combinations of QIV ± adjuvant ± LNPs and spleens were harvested at 10 DPV from all vaccinated as well as unvaccinated animals. Spleens were collected in 5 mL of RPMI-1640 media supplemented with 2% FBS and 1× penicillin/streptomycin and kept on ice. A single-cell suspension of splenocytes was obtained by homogenizing the spleens against a 70-μm strainer. Interferon gamma (IFN-γ) or interleukin-4 (IL-4) ELIspot assays were performed using 10^5^ splenocytes per well in a 96-well polyvinylidene difluoride (PVDF) ELIspot plates provided in the ELIspot kits (**Supplementary Table**), precoated with IFN-γ or IL-4 capture antibodies, respectively, according to the manufacturer’s protocol. Splenocytes were left unstimulated or restimulated either with hemagglutination (HA-H1N1) overlapping 15-mer peptides or whole live IVR-180 (H1N1) virus and incubated overnight in 37°C incubator. The wells were washed thrice with water to get rid of cells and incubated with 100 μL of biotinylated polyclonal detection antibody against IFN-γ or IL-4 for 1.5 h at RT, followed by an incubation with streptavidin-HRP-conjugated antibody for 1 h at RT. The plates were finally incubated with 100 μL of the substrate for 1 h in dark, followed by thorough washing under tap water multiple times. The plates were air-dried in the dark and the number of spots in each well was manually counted using a dissection microscope. The results were represented as number of IFN-γ- or IL-4-producing splenocytes per million splenocytes.

### Virus challenge

A high-titer dose of IVR-180 H1N1 virus of 18,000 PFU, which corresponds to 100× lethal dose that kills 50% of female BALB/c mice, per animal was used for intranasal infection in a final volume of 50 µL per mouse. The virus challenge was performed under mild anesthesia with ketamine/xylazine (intraperitoneal) as recommended by IACUC. The unvaccinated but challenged group was used as a control in the experiment. Body weights were recorded every day post-infection until lung harvest. The lungs were collected at 5 DPI in 500 μL of 1× PBS and homogenized using a tissue homogenizer. The lysate thus obtained was stored at −80°C for viral titrations.

### Plaque assay

Virus titrations were performed by plaque assays to quantify the replicating virus titers in the lungs of vaccinated versus unvaccinated mice. The lung homogenate (or lysate) was 10-fold serially diluted in 1× PBS and incubated on pre-seeded and pre-washed monolayers of MDCK cells for 1 h in an incubator, at 37°C, 5% CO_2_ with gentle shaking every 5 min. The diluted samples were then removed, and the monolayers were again briefly washed with 1 mL of 1× PBS. Lastly, 1 mL of the overlay mixture [2% oxoid agar and 2× minimal essential medium (MEM) supplemented with 1% diethyl-aminoethyl (DEAE)-dextran and 1 mg/mL tosylamide-2- phenylethyl chloromethyl ketone (TPCK)-treated trypsin] was added on top of the monolayers and incubated for 48 h in the incubator, at 37°C, 5% CO_2_. The plates were finally fixed in 4% formaldehyde. The overlay was removed, and the plaques were immune-stained with IVR-180-postchallenge polyclonal serum, diluted 1:1,000 in blocking buffer. The plates were washed and incubated with 1:5,000 dilution of HRP-conjugated anti-mouse secondary antibody for 1 h at RT with gentle shaking. Followed by a brief washing in 1× PBST, the plaques were finally visualized with True Blue substrate and the number of plaques was counted and presented as PFU/mL.

### Multiplex cytokine ELISA

Luminex-based cytokine ELISA was performed for simultaneous measurements of different cytokines in the lung homogenates from IVR-180-infected mice using the Th1/Th2 Cytokine 11-Plex Mouse ProcartaPlex™ kit (Invitrogen; EPX110-20820-901). Lungs were harvested at 5 DPI, homogenized in 500 μL of PBS, and centrifuged at 5,000 *g* for 5 min. Twenty-five microliters of each lysate was used for the assay. The following cytokines were measured: Granulocyte macrophage colony-stimulating factor (GMCSF), interleukin (IL)-1β, IL-4, IL-5, IL-6, IL-12p70, IL-13, IL-18, and interferon gamma (IFN-γ). The assay was performed according to the manufacturer’s guidelines and the readings were recorded using the Luminex 100/200 system.

### Software

The schematic figures were created with BioRender.com. GraphPad Prism version 10 was used for data visualization, analysis, graph plotting, and statistical analysis. Principal component analysis was performed using the statistical software package R: R Core Team (2023). R: A Language and Environment for Statistical Computing. R Foundation for Statistical Computing, Vienna, Austria. https://www.R-project.org/.

## Data availability statement

The raw data supporting the conclusions of this article will be made available by the authors, without undue reservation.

## Ethics statement

The animal study was approved by the Institutional Animal Care and Use Committee of the Icahn School of Medicine at Mount Sinai. The study was conducted in accordance with the local legislation and institutional requirements.

## Author contributions

SJ: Conceptualization, Investigation, Methodology, Validation, Visualization, Writing – original draft, Writing – review & editing. AL: Methodology, Validation, Writing – original draft, Writing – review & editing. GS: Investigation, Writing – review & editing. GL: Formal analysis, Investigation, Methodology, Validation, Writing – review & editing. YC: Methodology, Resources, Writing – review & editing. TY: Methodology, Resources, Writing – review & editing. AG-S: Funding acquisition, Writing – review & editing. BG: Formal analysis, Funding acquisition, Investigation, Methodology, Resources, Supervision, Writing – review & editing. MS: Conceptualization, Data curation, Formal analysis, Funding acquisition, Investigation, Methodology, Project administration, Resources, Software, Supervision, Validation, Visualization, Writing – original draft, Writing – review & editing.

## Supplementary material

Supplementary Figure 1 Chemical structure of in-house synthesized ionizable lipids- S-Ac7-Dog and K-Ac7-Dsa lipids, comprising a disulfide bond that can be cleaved by reduction and a ketal bond that can be cleaved by acidic pH, respectively.

Supplementary Figure 2 Heatmap showing mean OD 450 ELISA values (*n* = 5 per group) plotted against serum dilutions for total IgG, IgG1 and IgG2a. 6-8-week female BALB/c mice were vaccinated with QIV with and without IMDQ-PEG-Chol and formulated into empty or SDI-encapsulating S-Ac7-Dog or K-Ac7-Dsa LNPs. Serum was collected 3 weeks post-vaccination by submandibular bleed. Total IgG, IgG1 and IgG2a titers were quantified by ELISA with 3-fold serum dilutions starting with 1:100, for H1 HA specific antibodies.

Supplementary Figure 3 All lungs from unvaccinated and vaccinated animals were collected at 5 DPI and the cytokine levels were quantified by multiplex ELISA. Levels of **(A)** IL-1β, **(B)** IL-5, **(C)** IL-13, **(D)** TNF-α, **(E)** IL-12 p70, **(F)** IL-4, **(G)** IL-6, **(H)** IFN-γ, **(I)** IL-18 and **(J)** GMCSF, for *n* = 6 animals per group are represented as geometric mean ± geometric SD, where each data point corresponds to individual mouse. Statistical analysis was performed using one-way ANOVA with a Dunnett’s multiple comparison test. The *p*-values shown are calculated in reference to the virus-challenged unvaccinated group (denoted as PBS).
