# Supplementary figures and images for: Lipid nanoparticle composition for adjuvant formulation modulates disease after influenza virus infection in quadrivalent influenza vaccine vaccinated mice
Source: Front Immunol. 2024 Apr 22;15:1370564. doi: 10.3389/fimmu.2024.1370564 (PMC11070541; doi:10.3389/fimmu.2024.1370564)

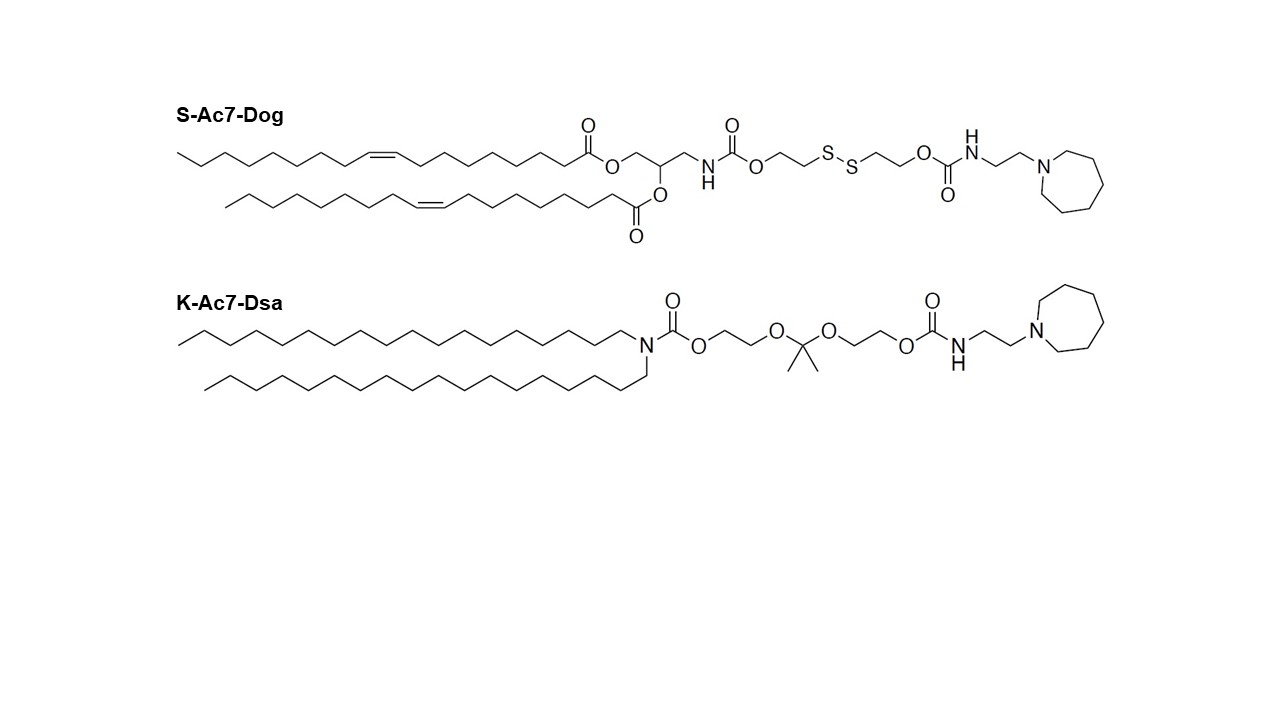

Supplement: Supplementary Figure 1 — Chemical structure of in-house synthesized ionizable lipids- S-Ac7-Dog and K-Ac7-Dsa lipids, comprising a disulfide bond that can be cleaved by reduction and a ketal bond that can be cleaved by acidic pH, respectively. [file Image_1.jpeg]

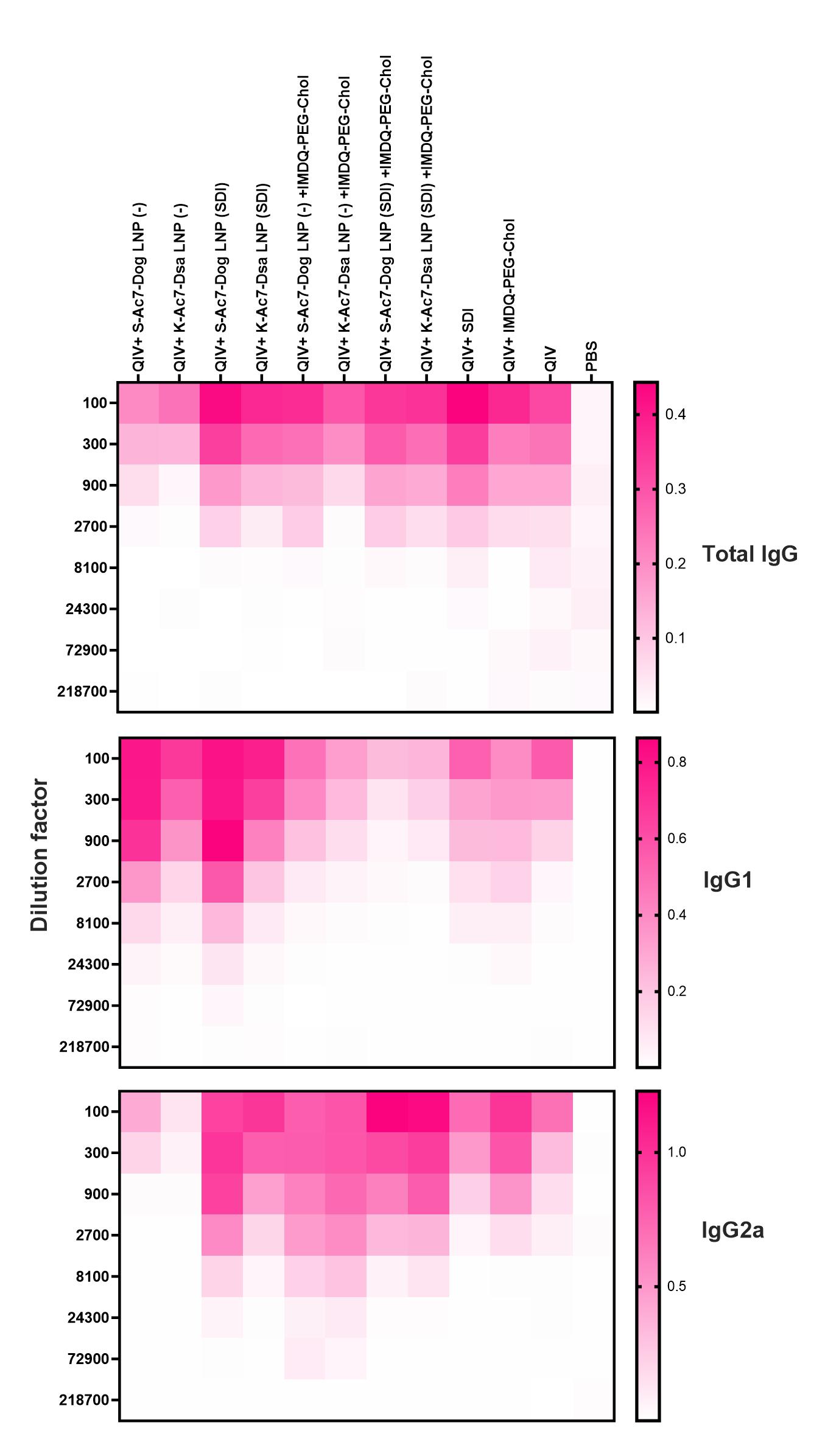

Supplement: Supplementary Figure 2 — Heatmap showing mean OD 450 ELISA values (n = 5 per group) plotted against serum dilutions for total IgG, IgG1 and IgG2a. 6-8-week female BALB/c mice were vaccinated with QIV with and without IMDQ-PEG-Chol and formulated into empty or SDI-encapsulating S-Ac7-Dog or K-Ac7-Dsa LNPs. Serum was collected 3 weeks post-vaccination by submandibular bleed. Total IgG, IgG1 and IgG2a titers were quantified by ELISA with 3-fold serum dilutions starting with 1:100, for H1 HA specific antibodies. [file Image_2.jpeg]

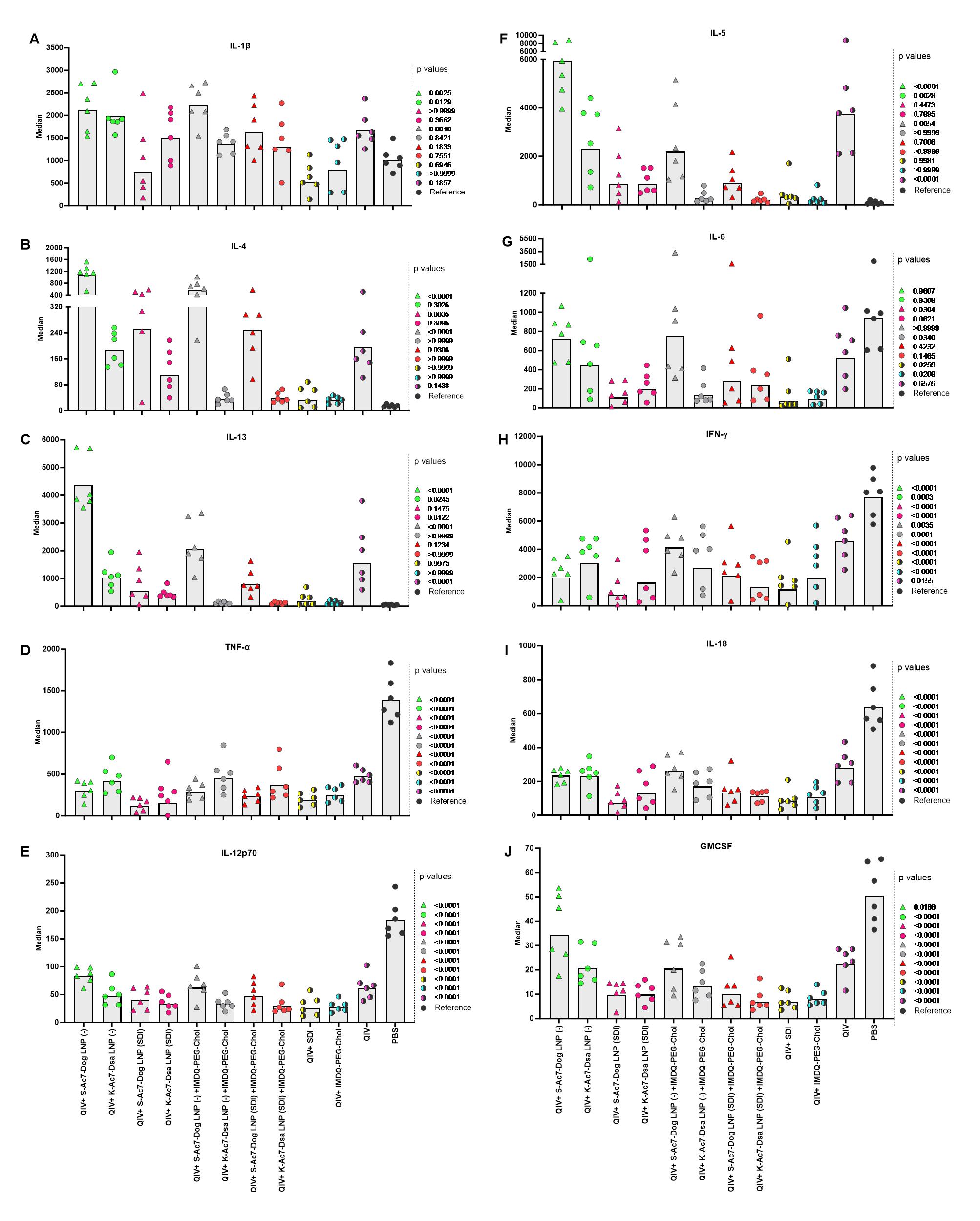

Supplement: Supplementary Figure 3 — All lungs from unvaccinated and vaccinated animals were collected at 5 DPI and the cytokine levels were quantified by multiplex ELISA. Levels of (A) IL-1β, (B) IL-5, (C) IL-13, (D) TNF-α, (E) IL-12 p70, (F) IL-4, (G) IL-6, (H) IFN-γ, (I) IL-18 and (J) GMCSF, for n = 6 animals per group are represented as geometric mean ± geometric SD, where each data point corresponds to individual mouse. Statistical analysis was performed using one-way ANOVA with a Dunnett’s multiple comparison test. The p-values shown are calculated in reference to the virus-challenged unvaccinated group (denoted as PBS). [file Image_3.jpeg]
