# Supplementary material for: Lipid nanoparticle composition for adjuvant formulation modulates disease after influenza virus infection in quadrivalent influenza vaccine vaccinated mice
Source: Front Immunol. 2024 Apr 22;15:1370564. doi: 10.3389/fimmu.2024.1370564 (PMC11070541; doi:10.3389/fimmu.2024.1370564)
Supplement: Supplementary file 4 [file Table_1.pdf]

Supplementary table: List of reagents and kits used in the study.

Reagent	Brand	Catalogue number
DMEM	Corning	10013-CV
RPMI 1640	Gibco	22400089
Penicillin/streptomycin	Corning	30002-CI
Goat HRP-conjugated secondary Anti-mouse IgG antibody	Abcam	Ab6823
Anti-mouse IgG1-HRP secondary antibody	Invitrogen	PA174421
Anti-mouse IgG2a-HRP secondary antibody	Invitrogen	A10685
TMB substrate	BD OptEIA	555214
KPL true blue substrate	Sera care	5510-0050
70µm strainer	BD	352340
Peptivator H1-HA peptide	MiltenyiBiotech	130-099-803
Oxoid agar	Thermofisher	LP0028B
EMEM	BioWhittaker	12684F
Receptor-destroying enzyme (RDE)	Hardy diagnostics	370013
TPCK	Sigma	T4376
10% methanol-free formaldehyde	Polysciences	040181
2M Sulfuric acid	Thermo fisher	S25898
Mouse IFN-γ ELISPOT kit	RnD systems	EL485
Mouse IL-4 ELISPOT kit	RnD systems	EL404
ELISA NUNC-maxisorp plates	Invitrogen	44240421
Th1/Th2 Cytokine 11-Plex Mouse ProcartaPlex™ Panel	Invitrogen	EPX110-20820-901
